# Post-contrast acute kidney injury – Part 1: Definition, clinical features, incidence, role of contrast medium and risk factors: Recommendations for updated ESUR Contrast Medium Safety Committee guidelines

**DOI:** 10.1007/s00330-017-5246-5

**Published:** 2018-02-09

**Authors:** Aart J. van der Molen, Peter Reimer, Ilona A. Dekkers, Georg Bongartz, Marie-France Bellin, Michele Bertolotto, Olivier Clement, Gertraud Heinz-Peer, Fulvio Stacul, Judith A. W. Webb, Henrik S. Thomsen

**Affiliations:** 1 Department of Radiology, C2-S, Leiden University Medical Center, Albinusdreef 2, NL-2333 ZA Leiden, The Netherlands; 2 Institute for Diagnostic and Interventional Radiology, Klinikum Karlsruhe, Academic Teaching Hospital of the University of Freiburg, Moltkestraße 90, D-76133 Karlsruhe, Germany; 3 Department of Diagnostic Radiology, University Hospitals of Basel, Petersgaben 4, CH-4033 Basel, Switzerland; 4 Service Central de Radiologie Hôpital Paul Brousse 14, av. P.-V.-Couturier, F-94807 Villejuif, France; 5 Department of Radiology, University of Trieste, Strada di Fiume 447, I-34149 Trieste, Italy; 6 Department of Radiology, Assistance Publique-Hôpitaux de Paris, Hôpital Européen Georges Pompidou, 20, rue Leblanc, Paris Cedex 15, F-71015 Paris, France; 7 Department of Radiology, Zentralinstitut für medizinische Radiologie, Diagnostik und Intervention, Landesklinikum St. Pölten, Propst Führer-Straße 4, AT-3100 St. Pölten, Austria; 8 S.C. Radiologia Ospedale Maggiore, Piazza Ospitale 1, I-34129 Trieste, Italy; 9 Department of Radiology, St. Bartholomew’s Hospital, University of London, West Smithfield, London, EC1A 7BE UK; 10 Department of Diagnostic Radiology 54E2, Copenhagen University Hospital Herlev, Herlev Ringvej 75, DK-2730 Herlev, Denmark

**Keywords:** Contrast media, Acute kidney injury, Glomerular filtration rate, Risk factors, Practice guidelines as topic

## Abstract

**Purpose:**

The Contrast Media Safety Committee (CMSC) of the European Society of Urogenital Radiology (ESUR) has updated its 2011 guidelines on the prevention of post-contrast acute kidney injury (PC-AKI). The results of the literature review and the recommendations based on it, which were used to prepare the new guidelines, are presented in two papers.

**Areas covered in part 1:**

Topics reviewed include the terminology used, the best way to measure eGFR, the definition of PC-AKI, and the risk factors for PC-AKI, including whether the risk with intravenous and intra-arterial contrast medium differs.

**Key Points:**

• *PC-AKI is the preferred term for renal function deterioration after contrast medium*.

• *PC-AKI has many possible causes*.

• *The risk of AKI caused by intravascular contrast medium has been overstated*.

• *Important patient risk factors for PC-AKI are CKD and dehydration*.

## Introduction

The Contrast Media Safety Committee (CMSC) of the European Society of Urogenital Radiology (ESUR) produced their most recent guidelines on what was then termed contrast induced nephropathy (CIN) in 2011 [1]. Guidelines on the use of contrast media (CM) in patients on dialysis and the use of CM in diabetic patients using metformin were published in 2002 and 2014 [2, 3]. This review provides the information to support the new CMSC guidelines, which were obtained using a structured literature review based on clinical questions and patient-intervention-comparator outcome (PICO) formatting. Since the literature related to the topics considered is so large, the results of the review have been split into two papers. The review only considers post-contrast kidney injury (PC-AKI) after intravascular iodine-based CM. Acute kidney injury (AKI) is not associated with intravascular gadolinium-based contrast agents in doses approved for clinical magnetic resonance imaging.

In this first paper on PC-AKI, the following topics related to diagnosis and risk are considered:

1. The clinical features and incidence of PC-AKI.
2. The choice of terms for renal function deterioration after CM, the degree of renal function deterioration used to diagnose PC-AKI, and the definitions of intravenous and intra-arterial CM administration.
3. The reliability of the various equations used to measure eGFR and the appropriate timing of eGFR measurement before CM administration.
4. The evidence that CM can cause AKI, the levels of renal function at which there is a risk of PC-AKI, and the recent evidence suggesting that the risk of PC-AKI may be lower after intravenous than after intra-arterial contrast medium.
5. The importance of the many risk factors for PC-AKI described in the literature.

Recommendations are made for items 2–5. The recommendations have been incorporated into version 10 of the ESUR CMSC guidelines (see Table 4 in Part 2).

## Clinical features and incidence of PC-AKI

The term PC-AKI is used to describe a decrease in renal function that follows intravascular administration of CM. The decrease in renal function is usually mild, peaking at 2–3 days, and renal function usually returns to baseline values within 1–3 weeks. Like all forms of AKI, an episode of PC-AKI is a marker for increased short- and long-term morbidity and mortality and prolonged hospital stay [4–10].

The risk of PC-AKI after intravenous (IV) CM has probably been overestimated. Two meta-analyses of 19,000 patients who had received IV CM showed PC-AKI incidences of 6.4 % (95 % CI 5.0–8.1) and 5.0 % (95 % CI 3.8–6.5) [11, 12]. In 1 % of all patients the decline in renal function persisted for 2 months, but the weighted incidence of renal replacement therapy (RRT) was as low as 0.06 % [11].

It has been suggested that intra-arterial (IA) CM administration during catheter-based angiography, with or without percutaneous coronary intervention (PCI), is associated with a higher incidence of PC-AKI than IV CM administration [13, 14]. However, there are many causes of AKI following angiography, and AKI may wrongly be attributed to the CM [15]. Catheter-based procedures may be complicated by haemodynamic instability, and by embolization of cholesterol or thrombi to the renal arteries caused by catheter manipulations [16]. Any of these may lead to post-interventional AKI, which is often misinterpreted as contrast-induced acute kidney injury (CI-AKI) [17, 18]. A large meta-analysis of cardiovascular outcomes after coronary angiography (CA) showed that the association between PC-AKI and mortality was strongly confounded by baseline clinical features that predisposed to both kidney injury and mortality [6]. The risk of PC-AKI reported in studies adjusted for confounding features was much lower than that from unadjusted studies. The incidence of AKI was 2.3 % and need for RRT 0.3 % in a recent retrospective analysis using propensity matching with controls of over 2,000 patients who had PCI [7].

## Materials and methods

The guidelines were developed using the Appraisal of Guidelines for Research and Evaluation (AGREE) II document [19]. A guideline Writing Group (WG) prepared ten clinical questions in Patient – Intervention – Comparison – Outcome (PICO) format [20]. Systematic search strings were developed with a professional librarian for four different biomedical literature databases (PubMed, Web of Science, Embase, and the Cochrane Library). Language was limited to English and German. Where necessary, additional systematic searches on specific topics, such as pediatric PC-AKI, were performed.

The titles and abstracts were screened for relevance and selected on predefined in- and exclusion criteria. Emphasis was put on comparative studies with strong scientific evidence, such as meta-analyses and systematic reviews, and prospective randomized controlled trials (RCTs). In addition, evidence was collected from comparative cohort, patient-control and non-comparative studies. Other important quality criteria were size of population studied, duration of follow-up and control for bias. Cross-referencing was used to find additional data. The four systematic searches for paper 1 yielded 3,086 references, of which 705 were selected from their title and abstract. The full texts of these 705 publications were reviewed and 105 were selected for inclusion in this paper. The quality of evidence was evaluated according to the Oxford Centre for Evidence Based Medicine (OCEBM) 2011 levels of evidence: Grade A: established scientific evidence; Grade B: scientific presumption; Grade C: low level of evidence [21]. Where there was no scientific evidence, recommendations were based on WG consensus and graded as expert opinion (Grade D).

Other factors such as availability of techniques or expertise, organizational consequences, financial costs and patient preferences were also considered. CM manuals and guidelines (American College of Radiology (ACR), Royal Australian and New Zealand College of Radiology (RANZCR), National Institute for Health and Care Excellence (NICE), Royal College of Radiologists (RCR), and Radiological Society of the Netherlands (RSTN)) were consulted where appropriate.

The recommendations prepared by the WG are the result of the available scientific evidence combined with these other sources of information. They were discussed at the CMSC meeting in Copenhagen, Denmark in February 2017 and the text of the final recommendations and guidelines was subsequently approved by the academic members of the CMSC. Once published in print, the validity of the CMSC guidelines will be routinely set at 6 years. However, the CMSC members constantly monitor the validity of the guidelines, and can propose revision at an earlier date if deemed necessary**.**

## Results

### QUESTION 1*:* What are the preferred terms and definitions to be used in PC-AKI?

#### Terminology

Until recently there has not been a generally accepted term for acute renal failure, which is a complex disorder with many possible causes and risk factors. Several nephrology groups, such as the Acute Dialysis Quality Initiative (ADQI) [22] and Kidney Disease: Improving Global Outcome (KDIGO) [23], have worked on finding a suitable term. The Acute Kidney Injury Network (AKIN), a group of experts in Critical Care and Nephrology, suggested Acute Kidney Injury (AKI) as the preferred term for acute renal failure to be used for all forms of AKI [24].

The CMSC recommends that the term PC-AKI should replace the older term of contrast-induced nephropathy (CIN) and suggests using the terms recommended by the ACR Committee on Drugs and Contrast Media [25] when AKI follows CM administration (Table 1). They state that *post-contrast acute kidney injury (PC-AKI)* is a general term that should be used if there is a sudden deterioration in renal function within 48 h of the intravascular administration of iodine-based CM. They describe PC-AKI as a *correlative* diagnosis. They recommend that the term *contrast-induced acute kidney injury (CI-AKI)* is reserved for cases where a causal relation can be shown between the administered CM and the deterioration in renal function. However, in clinical practice it is usually difficult to distinguish CI-AKI from PC-AKI and very few of the published studies have a suitable control group to allow the two conditions to be separated. Thus, many cases of PC-AKI seen in clinical practice or reported in clinical studies are likely to be coincident to, but not caused by, CM administration.

**Table 1:** PC-AKI: Terminology and definition

The preferred term for acute kidney injury associated with CM administration when no control population is available is *Post-Contrast Acute Kidney Injury (PC-AKI)*. The term *Contrast-Induced Acute Kidney Injury (CI-AKI)* should be used only when comparison with a control allows CM to be shown to be the cause of the acute kidney injury.
Level of Evidence D
PC-AKI and CI-AKI should be defined as an increase in sCr of ≥ 0.3mg/dl, or of ≥ 1.5–1.9 times baseline (KDIGO definition of AKI) in the 48–72 h following CM administration.
Level of Evidence C

#### Renal function definitions of PC-AKI

The diagnosis of PC-AKI is usually based on surrogate measures of absolute or relative change in serum creatinine (sCr), rather than patient outcomes, such as renal failure, need for RRT or mortality. The KDIGO Practice Guidelines [26, 27] adopted the older AKIN criteria [24] and recommended division of AKI into three stages dependent on sCr and/or urine output (Table 2).

**Table 2:** Acute Kidney Injury Staging (KDIGO) and CKD-EPI and Schwartz Equations for calculating eGFR

(a) KDIGO staging for AKI		
Stage	Serum creatinine	Urine output
1	sCr ≥ 0.3 mg/dl (≥ 26.5 μmol/L), or	< 0.5 ml/kg/h for 6–12h
	sCr increase of 1.5–1.9 x baseline	
2	sCr increase of 2.0–2.9 x baseline	< 0.5 ml/kg/h for ≥ 12h
3	sCr ≥ 4.0 mg/dl (≥ 354 μmol/L) or	< 0.3 ml/kg/h for ≥ 24h
	sCr increase > 3.0x baseline	or Anuria for ≥ 12h
	or need for renal replacement therapy	
(b) CKD-EPI equation (sCr in μmol/L; age in years).		
eGFR (ml/min/1.73 m^2^) =		
Female sCr ≤ 62 μmol/L:	144 x (sCr / 62)^-0.329^ x 0.993^Age^	
Female sCr > 62 μmol/L:	144 x (sCr / 62)^-1.209^ x 0.993^Age^	
Male sCr ≤ 80 μmol/L:	141 x (sCr / 80)^-0.411^ x 0.993^Age^	
Male sCr > 80 μmol/L:	141 x (sCr / 80)^-1.209^ x 0.993^Age^	
*All equations x 1.159 if African American race*		
(c) Revised Schwartz equation (sCr in μmol/L; patient length in cm).		
eGFR (ml/min/1.73 m2) =	36.5 × Length / sCr	

The ESUR CMSC defined Contrast-Induced Nephropathy (CIN) in their first survey-based guideline as “a condition in which an impairment in renal function (an increase in sCr by more than 25 % or 44 μmol/L, or 0.5 mg/dl) occurs within 3 days following the intravascular administration of a contrast medium *in the absence of an alternative aetiology”* [28].

Multiple studies have shown that the incidence of PC-AKI is largely dependent on the definition used [29–31]. A relative increase in sCr of > 25 % has been the most sensitive indicator, with absolute values being less sensitive. In coronary angiography studies, relative definitions had more prognostic relevance [29]. In other studies, however, relative increases in sCr were found to overestimate PC-AKI and absolute values were considered preferable [32]. Relative values seem to be more sensitive for patients with CKD 3B (eGFR 30–44 ml/min/1.73m^2^) and CKD 2 (eGFR 60–89 ml/min//1.73m^2^), and absolute values seem to be more sensitive for patients with CKD 3A (eGFR 45–59 ml/min/1.73m^2^) [33, 34]. Studies in critically ill populations using the AKIN definition found that development of AKI correlated with ICU mortality [35].

The KDIGO criteria are more rigorously derived than the CIN definition and are now being adopted as the standard for PC-AKI studies [36]. The CMSC, like the European Renal Best Practice (ERBP) working group, recommends that the definition of PC- AKI (or CI-AKI) should use the KDIGO definition of AKI: an increase in sCr of ≥ 0.3 mg/dl, or a sCr increase of ≥ 1.5– 1.9 times baseline [37, 38] (Table 1). The KDIGO recommendation is that the renal function change should be within 48 h, but the CMSC recommends retaining a period of 48–72 h after CM as being more practical for diagnosing PC-AKI in radiological practice, the majority of which involves outpatients.

#### Intravenous and direct and indirect intra-arterial CM administration: definition of terms

The term *intravenous CM administration* indicates that CM reaches the renal arteries after dilution by circulation through the right heart and pulmonary circulation or a systemic capillary bed.

The same is true for *intra-arterial CM administration with second-pass renal exposure*, such as via catheters into the right heart and pulmonary arteries and via catheters directly in the carotid, subclavian, brachial, coronary and mesenteric arteries, as well as into the infrarenal aorta and the iliac, femoral and crural arteries. *Note:* Because of backflow during this type of IA injection, small doses of CM may reach the kidney in a relatively undiluted form.

The term i*ntra-arterial CM administration with first-pass renal exposure* indicates that CM reaches the renal arteries during its first pass in a relatively undiluted form, depending on the distance of the site of injection from the renal arteries. This occurs with injections through catheters into the left heart, the thoracic and suprarenal abdominal aorta, and selectively into the renal arteries. *Note*: In suprarenal aortic injections, some of the injected CM escapes via suprarenal aortic side-branches and reaches the kidney after dilution in the circulation.

### QUESTION 2*:* What are the best equations for GFR estimation in European populations?

Total glomerular filtration rate (GFR) is considered the best overall index of kidney function, but cannot be measured easily in clinical practice, so GFR is estimated using sCr as an endogenous filtration marker. In 1999, the Modification of Diet in Renal Disease (MDRD) equation [39] was introduced for estimating GFR. The quality of GFR estimates largely depends on the accuracy of the creatinine measurements, and should be based on sCr assays standardized to reference methods [40]. The MDRD equation has therefore been re-expressed for use with sCr assays standardized using isotope dilution mass spectroscopy (IDMS) [41].

In 2009, the CKD-EPI equation was proposed by the Chronic Kidney Disease Epidemiology Collaboration (CKD-EPI), and was shown to be superior to the MDRD equation, especially at higher GFRs (Table 2) [42]. The National Kidney Foundation recommended replacing the MDRD by the CKD-EPI equation for routine clinical use [43]. The CMSC therefore recommends the CKD-EPI equation for routine use in adults (Table 3). All creatinine-based equations should be used with caution in people with abnormally high or low muscle mass. Caution should also be exercised in patients with AKI, because sCr takes several days to stabilize and may not reflect current GFR.

**Table 3:** Formulae for eGFR and timing of eGFR measurement

The CKD-EPI equation for estimated GFR (eGFR) is recommended for adults. As with all creatinine-based eGFRs, results should be interpreted with caution in people with abnormally high or low muscle mass
Level of Evidence A
The revised Schwartz formula (2009) for eGFR is recommended for children. As with all creatinine-based eGFRs, results should be interpreted with caution in people with abnormally high or low muscle mass
Level of Evidence C
eGFR is not reliable in patients with known AKI
Level of Evidence A
The CMSC considers eGFR measurements before intravascular CM exposure valid for *a maximum of:*
1) 7 days* if the patient has (a) an acute disease, an acute deterioration of a known chronic disease or any other adverse event that could have negatively influenced renal function (eGFR), or (b) is a hospital inpatient
2) 3 months (a) if the patient has a chronic disease with stable renal function (eGFR) and (b) in all other patients
Level of Evidence D

*Note: in patients with AKI it is advisable to monitor eGFR frequently, so a maximum of 1-2 days may be advisable.

There are other equations for specific subgroups, such as the Lund-Malmö (LM) revised equation for the Swedish population [44], the Berlin Initiative Study (BIS-1) equation for the elderly German population [45], and the full age spectrum (FAS) equation for children and adults [46]. However, these equations have not been validated in other populations. Cystatin C equations for estimation of GFR may have advantages over sCr-based equations but are limited by additional costs and lack of an international reference system for calibration [47].

#### Estimation of GFR in children

When estimating GFR in children, sCr levels should be measured by standardized reference methods because serum concentrations are lower than in adults [48]. The CMSC therefore recommends the revised Schwartz equation for routine clinical use in children (Table 3). The widely-used Schwartz equation [49] was revised in 2009 to include the IDMS method and plasma iohexol clearance as standardized reference methods [50] (Tables 2 and 3).

A Cystatin C-based equation has been proposed that showed the best accuracy (91 %) when combined with height/SCr, height, sex and blood urea nitrogen (BUN) [49]. However, this requires an additional BUN, which lacks standardized measurement, and Cystatin C requires standardization and calibration [51]. In children with increased muscle mass both the sCr and Cystatin C based Schwartz formulas tend to overestimate GFR.

#### Point-of-care creatinine measurements

Point-of-care (PoC) whole blood creatinine may be measured with the older Jaffe (alkaline picrate) method or by enzymatic methods, with the latter considered more accurate. Although such measurements have practical advantages in patients with increased risk of PC-AKI, PoC devices may lead to overestimation of renal function in severe kidney failure with resultant incorrect risk stratification [52]. Laboratory professionals should be consulted about analytical performance and quality assurance of whole blood PoC creatinine measurement.

#### For how long do GFR estimations remain valid?

There are no studies available on how long eGFR measurements remain valid for estimating the PC-AKI risk. The eGFR measurements can be regarded as stable in individuals without CKD or underlying co-morbidities such as heart failure or hypertension who are not taking nephrotoxic drugs.

The CMSC considers eGFR measurements before intravascular CM exposure valid for *a maximum of:*

1. 7 days* if the patient
  - has an acute disease, an acute deterioration of a known chronic disease or any other adverse event that could have negatively influenced renal function (eGFR), or
  - is a hospital inpatient
2. 3 months
  - if the patient has a chronic disease with stable renal function (eGFR), and
  - in all other patients (Table 3)

*Note: In patients with AKI, eGFR should be monitored frequently, and a maximum of 1–*2 days is advisable.*

### QUESTION 3: What is the evidence that contrast media are truly a causative factor in AKI and what are the eGFR values below which there is a risk of PC-AKI?

Contrast-induced nephropathy was accepted for many years, but more recently it has been questioned whether CM causes the deterioration in renal function that may occur after CM administration [17, 53]. There are important limitations in many studies that assess whether CM causes AKI. Most studies evaluate the use of IA CM in CA and/or percutaneous coronary intervention (PCI) in patients with significant co-morbidities and therefore may not be relevant for intravenous administration, and most studies do not have adequate control groups [54, 55].

#### Intravenous CM administration

There is controversy about the causal relationship between exposure to IV CM and PC-AKI, since there are no prospective RCTs confirming this association [56, 57]. Without controlled studies, many factors such as diet, hydration, physiological variation in sCr over time, and a variety of nephrotoxic risk factors, including medications, which may influence renal function, cannot be distinguished from any effect of the CM [17, 18, 58]. Although RCTs have the strongest research design for assessing the effects of interventions, assessment of rare conditions such as PC-AKI by RCT would require large numbers of patients [53].

Based on comparisons of the relatively few studies with and without control populations it has been suggested that the risk of PC-AKI after IV CM has been overestimated [53, 59]. A meta-analysis that retrospectively studied 13 non-randomised controlled studies was unable to find an increased incidence of AKI in patients who received intravenous contrast medium [60].

Evidence from observational studies may need to be used, despite the recognised methodological problems [61]. Recently, a few large-scale studies using propensity score (PS)-matching for the evaluation of PC-AKI in patients undergoing contrast-enhanced CT, which stratified subjects according to their baseline sCr or eGFR, have been published [62–65]. These studies were unable to identify a risk of PC-AKI in patients with eGFR ≥ 30 ml/min/1.73m^2^, but there is conflicting evidence on whether patients with severe renal impairment (eGFR <30 ml/min/1.73m^2^) are at increased risk of PC-AKI [63, 65]. Lack of information on hydration status was a limitation in these studies, but when hydration status was added to an improved PS model, the findings were similar [66]. The failure to adjust for the various predictor variables in previous observational studies may explain the differences between them and the recent PS matching-based studies. Remaining major limitations of observational studies are the low numbers of patients with severe renal impairment, and the variability of data available on, for example, prophylactic volume expansion and the CM dose administered.

#### Comparison of intra-arterial and intravenous CM administration in the same patients

A limited number of studies have directly compared IV to IA CM administration, using the patient as their own control. The risk of PC-AKI as well as its clinical course was independent of the route of administration in four retrospective studies of patient populations with varying degrees of renal impairment [67–70], and PC-AKI rates were similar to the rates for unenhanced CT [70]. However, these studies suffer from selection bias and procedures with IA CM administration with first- and second-pass renal exposure were not separated.

#### Intra-arterial CM administration

The PC-AKI incidence following direct IA CM administration with first-pass renal exposure is frequently reported to be higher than after IV administration, but this remains controversial [71, 72]. Problems with confounding factors are most significant in studies on patients that undergo catheter-based CA and/or PCI because it is impossible to separate the effects of contrast media from the effects of co-morbidity, catheter manipulations or other procedural variables. In large meta-analyses on cardiovascular outcome the PC-AKI incidence may have been strongly confounded by baseline clinical characteristics, both for first- and second-pass IA CM administration [6, 73]. Nonetheless, AKI in general is a significant problem in these patients and is associated with increased morbidity, longer length of hospital stay and higher cost [74], and may be associated with mortality in a significant percentage of cardiac patients [7]. Second-pass IA CM administration is considered to have no higher risk than IV CM administration.

Since it is difficult to separate the effects of the procedure from those of the CM, the CMSC decided, for optimal safety, to choose a higher cut-off eGFR level for preventive measures in patients undergoing catheter-based diagnostic or interventional examinations using IA CM administration with first-pass renal exposure, even though some of the risk may relate to the procedure. Also, the CMSC decided to include CA and/or PCI in this category because these examinations frequently combine IA CM administration with both first- and second-pass renal exposure (Table 4).

**Table 4:** Risk of PC-AKI

(a) Levels of eGFR at which there is a risk
The risk of PC-AKI in patients with eGFR ≥ 30 ml/min/1.73m^2^ after intravenous and intra-arterial CM administration with second-pass renal exposure is very low, but there is conflicting evidence on the risk for intra-arterial CM administration with first-pass renal exposure
Level of Evidence: B
Preventive measures are recommended for patients with eGFR < 30 ml/min/1.73m^2^ before intravenous and intra-arterial CM administration with second-pass renal exposure
Level of Evidence: C
Preventive measures are recommended for patients with eGFR < 45 ml/min/1.73m^2^ if they are in ICU or if they will receive intra-arterial CM administration with first-pass renal exposure
Level of Evidence: C
Recommendations for prevention of PC-AKI in adults may also be used in children and adolescents
Level of Evidence D
(b) Risk factors
The principal risk factor for PC-AKI is impaired renal function. Most other published patient-related risk factors are risk factors for the presence of chronic kidney disease or AKI, and are not specific for PC-AKI
Level of Evidence B
There is no difference in PC-AKI risk between IOCM and LOCM. The use of ionic, high-osmolar CM and repeated CM injections in a short period (48–72 h) should be avoided
Level of Evidence C
When CM are injected intravenously, there is insufficient evidence that CM dose is a risk factor. When CM are injected intra-arterially, the ratio of CM dose (in gram Iodine) / *absolute* eGFR (in ml/min) should be kept below 1.1 or the ratio of CM volume (in ml) / eGFR (in ml/min/1.73m^2^) should be kept below 3.0 when using a CM concentration of 350 mgl/ml
Level of Evidence C

#### Special populations

There is limited evidence about PC-AKI in several special populations, such as patients with renal or renal and pancreatic transplants, or critically ill patients. In renal transplant recipients, the incidence of PC-AKI in patients receiving either IV or IA CM was not higher than in patients without transplants, and there was no graft loss or need for dialysis [75–77]. Critically ill patients in ICU with multi-organ disease have a greater risk profile for AKI than other inpatients, and AKI incidence varies with subpopulation, study design and hydration status [33, 78, 79]. Without properly controlled studies, it is impossible to know the role of CM in causing the AKI. Although earlier studies failed to show a role of CM [80, 81], a recent large PS-matched controlled study suggested an increased PC-AKI risk for ICU patients with eGFR < 45 ml/min/1.73m^2^ [82].

#### Paediatric PC-AKI

There are very few studies on paediatric PC-AKI [83–85]. As the incidence of PC-AKI seems similar in children and adolescents to that in adults, the CMSC considers that for optimal safety the recommendations for sCr determination and prevention of PC-AKI, which are predominantly based on studies in adults (aged 18+ years), should also be used for children and adolescents (Table 4).

### QUESTION 4: What are the patient- and procedure-related risk factors for developing PC-AKI and which patient populations have a higher risk for developing PC-AKI?

#### Patient-related risk factors

Impaired renal function is the most important patient risk factor for PC-AKI. Many meta-analyses and systematic reviews of uncontrolled studies have identified a wealth of possible clinical risk factors for AKI in general such as old age, female gender, low BMI, classic cardiovascular and metabolic risk factors, malignancy, inflammation, bleeding, anaemia and hyperuricaemia [11, 12, 86–96]. However, uncontrolled studies cannot reliably differentiate baseline clinical risk factors from effects attributable specifically to CM. In a meta-analysis of controlled studies, no additional risk factors specific for CM were demonstrated [60] (Table 4). The effect of two or more risk factors is additive and increases the risk of PC-AKI.

#### Procedure-related risk factors: CM type and dose

A variety of risk factors have been related to the type of CM and the way it is administered.

Multiple meta-analyses have shown no evidence that iso-osmolar CM (IOCM) are associated with a significantly lower rate of PC-AKI than non-ionic, low osmolar CM agents (LOCM) [97–100]. However, the risk of PC-AKI is increased when ionic, high-osmolar CM are used [101]. Repeated CM administration within a short interval (48–72 h) has been shown to increase the risk of PC-AKI [86] (Table 4).

Evidence about the influence of CM dose (CM volume x CM concentration) indicates dependence on the route of administration. There is insufficient evidence that dose is a problem with IV CM. However, for direct IA CM administration in coronary angiographic intervention it is advisable to keep the ratio of CM dose (in grams Iodine) to *absolute* eGFR (in ml/min; corrected for body surface area) below 1.1 [102, 103] or to keep the ratio of CM volume (in ml) to eGFR (in ml/min/1.73m^2^) below 3.0 when using a CM concentration of 350 mgl/ml [104, 105] (Table 4).

## Conclusion

PC-AKI has been adopted as the best term to apply to renal function deterioration after intravascular CM administration because, unlike some of the older terms, it does not imply that CM is the cause. Stage 1 of the KDIGO classification of AKI is recommended as the change in renal function used to diagnose PC-AKI. The principal risk factor for PC-AKI is impaired renal function, and the recommended ways to measure this are by the CKD-EPI equation in adults and the Schwartz equation in children. In recent years, it has become apparent that the risk of true CI-AKI was overstated in the past. When properly corrected for the many other possible causes of AKI in patients with chronic kidney disease, the risk of CI-AKI when modern low osmolar CM are administered IV or IA is low. Repeated CM administration within a 24- to 48-h period increases the risk of CI-AKI. The evidence of a higher risk with IA than with IV CM administration is limited, but the CMSC nonetheless considers that the cut-off levels of eGFR used to indicate the need for prophylaxis before IA administration with first-pass renal exposure should be stricter, and that there should be a maximum volume of CM given intra-arterially during any examination or procedure with first-pass renal exposure.

The recommendations made in this paper have been incorporated into the ESUR CMSC guidelines (see Table 4, Part 2).

## Abbreviations

ACR: American College of Radiology
ADQI: Acute Dialysis Quality Initiative
AGREE: Appraisal of Guidelines for Research and Evaluation
AKI: Acute kidney injury
AKIN: Acute Kidney Injury Network
BIS: Berlin Initiative Study
BUN: Blood urea nitrogen
CA: Coronary angiography
CI: Confidence interval
CI-AKI: Contrast-induced acute kidney injury
CIN: Contrast-induced nephropathy
CKD: Chronic kidney disease
CKD-EPI: Chronic Kidney Disease Epidemiology Collaboration
CM: Contrast media
CMSC: Contrast Media Safety Committee
CT: Computed tomography
eGFR: Estimated glomerular filtration rate
EBM: Evidence-based medicine
ERBP: European Renal Best Practice
ESUR: European Society of Urogenital Radiology
FAS: Full age spectrum
GFR: Glomerular filtration rate
IA: Intra-arterial
ICU: Intensive care unit
IDMS: Isotope Dilution Mass Spectroscopy
IV: Intravenous
KDIGO: Kidney Disease Improving Global Outcome
LM: Lund Malmö
MDRD: Modification of Diet in Renal Disease
NICE: National Institute for Health and Care Excellence
OCEBM: Oxford Center for Evidence Based Medicine
PC-AKI: Post-contrast acute kidney injury
PCI: Percutaneous coronary intervention
PICO: Patient-Intervention-Comparator-Outcome
PoC: Point of Care
PS: Propensity Score
RANZCR: Royal Australian and New Zealand College of Radiology
RCR: Royal College of Radiologists
RCT: Randomised controlled trials
RRT: Renal replacement therapy
RSTN: Radiological Society of The Netherlands
sCr: Serum creatinine
WG: Writing Group
